# CD56 polysialylation promotes the tumorigenesis and progression via the Hedgehog and Wnt/β-catenin signaling pathways in clear cell renal cell carcinoma

**DOI:** 10.1186/s12935-023-03165-5

**Published:** 2023-12-12

**Authors:** Yuli Jian, Lin Zhang, Li Gong, Mengting Ding, Xiaoxin Sun, Xiao Yu, Shaohui Lv, Jinjing Li, Deyong Yang, Shujing Wang

**Affiliations:** 1 Liaoning Provincial Core Lab of Glycobiology and Glycoengineering, College of Basic Medical Sciences, Dalian Medical University, Dalian, 116044 China; 2 Department of Urology, First Affiliated Hospital of Dalian Medical University, Dalian, 116011 China; 3 Department of Pathology and Forensic Medicine, College of Basic Medical Sciences, Dalian Medical University, Dalian, 116044 China

**Keywords:** PSA-CD56, Clear cell renal cell carcinoma, Hedgehog, Wnt/β-catenin

## Abstract

**Background:**

CD56 has been observed in malignant tumours exhibiting neuronal or neuroendocrine differentiation, such as breast cancer, small-cell lung cancer, and neuroblastoma. Abnormal glycosylation modifications are thought to play a role in regulating tumour cell proliferation, migration, and invasion. Nevertheless, the exact roles and molecular mechanisms of CD56 and polysialylated CD56 (PSA-CD56) in the development and progression of clear cell renal cell carcinoma (ccRCC) remain elusive. Here we unveil the biological significance of CD56 and PSA-CD56 in ccRCC.

**Methods:**

In this study, we employed various techniques, including immunohistochemistry (IHC), RT-qPCR, and western blot, to examine the mRNA and protein expression levels in both human ccRCC tissue and cell lines. Lentivirus infection and CRISPR/Cas9 system were utilized to generate overexpression and knockout cell lines of CD56. Additionally, we conducted several functional assays, such as CCK-8, colony formation, cell scratch, and transwell assays to evaluate cell growth, proliferation, migration, and invasion. Furthermore, we established a xenograft tumor model to investigate the role of CD56 in ccRCC in vivo. To gain further insights into the molecular mechanisms associated with CD56, we employed the Hedgehog inhibitor JK184 and the β-catenin inhibitor Prodigiosin.

**Results:**

CD56 was significantly overexpressed in both human ccRCC tissues and renal cancer cell lines compared to adjacent normal tissues and normal renal epithelial cells. In vitro and in vivo experiments revealed that the knockout of CD56 inhibited the proliferation, migration, and invasion capabilities of ccRCC cells, whereas the overexpression of PSA-CD56 promoted these capacities. Finally, PSA-CD56 overexpression was found to activate both the Hedgehog and Wnt/β-catenin signaling pathways.

**Conclusion:**

Our findings demonstrate that the oncogenic function of CD56 polysialylation plays a vital role in the tumorigenesis and progression of ccRCC, implying that targeting PSA-CD56 might be a feasible treatment target for ccRCC.

**Supplementary Information:**

The online version contains supplementary material available at 10.1186/s12935-023-03165-5.

## Introduction

Renal cell carcinoma (RCC) is one of the malignancies that affect the urinary system and is extensively researched due to its high degree of heterogeneity [1]. RCC comprises various subtypes, with clear cell RCC (ccRCC) constituting approximately 75% of cases. The remaining subtypes include papillary RCC (pRCC) at 15%, chromophobe RCC (chRCC) at 5%, and unclassified RCC (uRCC) at 4% [2]. Despite the considerable extension of progression-free survival (PFS) in patients with ccRCC through surgical intervention, approximately 30% of patients continue to experience metastasis, resulting in a bleak prognosis. Moreover, these patients exhibit insensitivity to conventional radiotherapy and chemotherapy following surgical procedures [3]. Consequently, there exists a pressing demand for biomarkers that can effectively diagnose pathological characteristics and subsequently enhance the prognostic outcomes of ccRCC patients, particularly in the targeted therapy era [4].

Neural cell adhesion molecule-1 (*NCAM1*), also known as CD56, is a glycoprotein found on the cell membrane that is used to diagnose lymphohematopoietic disorders [5]. The mRNA of CD56 undergoes various splicing mechanisms, resulting in the production of three distinct molecular subtypes with molecular weights of 120, 140, and 180 kDa, respectively [6]. Research indicates that the 120 kDa variant is a glycosylphosphatidylinositol (GPI)-anchored protein, while the 140 kDa and 180 kDa variants are transmembrane proteins. The 140 kDa and 180 kDa CD56 proteins are predominantly present in undifferentiated tissues or malignant tumour cells [7]. Studies have shown that CD56 exhibits high levels of expression in nerve cells, various neuroendocrine tumours and tumor-infiltrating lymphocytes [8]. Consequently, it has become the most commonly employed immunomarker for the diagnosis of small-cell lung tumours [9]. Notably, previous reports demonstrated that highly expressed CD56 in ameloblastoma suppressed the migration of ameloblastoma cells, whereas CD56 knockdown impede the proliferation, migration and epithelial-to-mesenchymal transition (EMT) of human melanoma cells, but enhance their apoptosis and autophagy [10, 11]. Malignant lymphomas of T-NK cell origin bear CD56, as well as multiple myeloma, melanoma and some cancers of epithelial origin, which suggests that CD56 is involved in tumor biology in some unknown manner [12].

Researchers discovered six potential *N*-glycosylation sites on CD56’s third, fourth, and fifth Ig-like domains, and the *NCAM1* gene is translated and transcribed at the end of the glycan chain of the fifth Ig-like domain by polysialyltransferases to form polysialylated CD56, namely PSA-CD56 [13, 14]. As stated in one study, the reduction of polysialic acid has been observed to decrease the migration of tumour cells, while simultaneously increasing the number of focal adhesions in a manner that is dependent on cell–cell contact and *NCAM* [15]. Moreover, polysialylation of NCAM-140 enhanced cell migration in a polysialyltransferase-specific manner [16]. The aforementioned studies indicate that CD56 and PSA-CD56 may have a significant impact on the initiation and progression of tumours, making them potential candidates for diagnostic and therapeutic purposes in the management of malignant diseases.

In this study, we presented findings that demonstrate a strong association between elevated levels of CD56 expression and clinical advancement as well as unfavourable prognosis in patients with ccRCC. Additionally, we observed a positive correlation between CD56 expression levels and the severity of pathological grades. To further investigate the underlying molecular mechanisms, we established knockout (KO) of CD56 in 786-O cells and overexpression (OE) of PSA-CD56 in Caki-1 cells. By elucidating the mechanisms by which CD56 and PSA-CD56 influence ccRCC progression, we hope to contribute to the development of targeted therapies and improved diagnostic strategies for patients with ccRCC.

## Materials and methods

### Patient cohort

Human kidney cancer tissues (n = 40) were obtained from ccRCC patients undergoing surgical resections at the First Affiliated Hospital of Dalian Medical University (Dalian, China) between January 2016 to December 2016 and the tumour grades of these tissues were confirmed by two pathologists (detailed clinicopathological features summarized in Table 1). The diagnose and imaging of ccRCC was based on ultrasonography, computed tomography and magnetic resonance imaging [17]. In addition, we purchased the tissue microarrays (HKidCRCC060PG01) to obtain the normal kidney tissues (n = 16) from Shanghai Outdo Biotech Company.

**Table 1:** Correlation between CD56 expression and the clinicopathological characteristics of ccRCC patients

Clinicopathologic characteristics	Group	NO. of cases (n = 40) (100%)	CD56 expression		*P*-value
			Low	High	
Age (years)	< 65	31 (77.5%)	17	14	0.5825
	≥ 65	9 (22.5%)	4	5	
Gender	Male	31 (77.5%)	17	14	0.5825
	Female	9 (22.5%)	4	5	
Tumor sizes	< 5 cm	19 (47.5%)	13	6	0.0551
	≥ 5 cm	21 (52.5%)	8	13	
Fuhrman grade	I-II	25 (62.5%)	18	7	**0.0014**
	III-IV	15 (37.5%)	3	12	

Bold represents that the value is statistically significant

All mRNA expression data of ccRCC patients and normal tissues were downloaded from The Cancer Genome Atlas (TCGA) (https://cancergenome.nih.gov/) and the Gene Expression Omnibus (GEO) (https://www.ncbi.nlm.nih.gov/geo/) databases. The TCGA, GSE53757, GSE68417, GSE105261, GSE36895, GSE46699, GSE71963, GSE76351, GSE15641, GSE53000 and GSE40435 datasets were analyzed by the package “DESeq2” or “limma” in R 4.2.2 software.

### Cell culture

Human ccRCC cell lines Caki-1, 786-O and A-498, and human normal renal epithelial cell line HK-2 were purchased from the Cell Bank of the Shanghai Life Science Institution, Chinese Academy of Sciences (Shanghai, China). Caki-1 cells were cultured in McCoy’s 5A medium (Macgene) while 786-O cells were cultured in RPMI 1640 medium (Gibco, Novato, CA, United States). HK-2 and A-498 cells have been cultured in MEM medium (KeyGEN BioTECH, China). The medium was supplemented with 10% fetal bovine serum (FBS, ExCell Bio, China) and 100 U/ml penicillin–streptomycin (Seven, China), and maintained in a cell incubator (Thermo Fisher Scientific, United States) at 37 °C with 5% CO_2_. All cell lines used in this study were confirmed to be mycoplasma negative every 3 months and authenticated by short tandem repeat (STR) profiling.

### Generation of CD56 overexpressed Caki-1 cell

In order to establish an overexpression-CD56 Caki-1 cell line, a targeted sequence designed to modulate the expression of the human *NCAM1* gene (Gene ID: 4684) was introduced into the pLenti-GIII-CMV-GFP-2A-Puro vector (ABM). Following the verification of the insert sequences through DNA sequencing, the Caki-1 cells were transfected with lentiviral fluid. 48 h after transfection, positive cells were selected by puromycin (5 μg/ml) for 7 days and then a single cell was isolated by limited dilution in a 96-well plate.

### Establishment of CD56 knockout 786-O cell

The CRISPR gene editing technique was employed to delete CD56 in the 786-O cell line. A single sgRNA consisting of 20 bases (5ʹ-GCCTTCGGCATCGCACACCA-3ʹ) was specifically designed for targeting the *NCAM1* gene. A U6-sgRNA-SFFV-Cas9-2A-Puro vector (ABM) containing an NCAM1-specific gRNA sequence was incorporated into the 786-O cell line through nucleofection. The selection of positive cells was carried out using puromycin at a concentration of 2 μg/ml for 7 days. Subsequently, a single cell was isolated through limited dilution in a 96-well plate. Following the isolation of novel individual clones, they were subsequently propagated and subjected to analysis through the utilization of RT-qPCR and western blot techniques.

### Hematoxylin and eosin (H&E) and immunohistochemistry analysis (IHC)

The tissues were prepared for serial sections after embedded. After using xylene and gradient alcohol, hematoxylin was used to stain the nucleus to dark blue and then the sections were differentiated several seconds in 1% of alcohol hydrochloride. The eosin solution was used to stain proteins to red to pink and dehydrated with gradient alcohol.

For IHC, tissue samples were fixed overnight in 4% paraformaldehyde in order to obtain paraffin-embedded sections. The sections were deparaffinized with xylene and rehydrated using gradient alcohol. After repairing the antigen with Tris–EDTA buffer (pH = 9.0) or citrate buffer, remove endogenous peroxidase by treating the slides with endogenous catalase blocker (3%). The tissues were then incubated with primary antibodies overnight at 4 °C. The next day, it was incubated with the enzyme-labelled goat anti-rabbit/mouse IgG polymer, followed by treatement with Diaminobenzidine (DAB). Finally, stained with hematoxylin and dehydrated with gradient alcohol and observed under the microscope. The antibodies were used: NCAM1/CD56 (1:10,000, Proteintech, 14255-1-AP), SUFU (1:100, Cell Signaling Technology, 2520), β-catenin (1:100, Proteintech, 66379-1-Ig), c-Myc (1:100, Bioworld, BS2462). The staining intensity was scored as 0 (no staining), 1 (weak staining), 2 (moderate staining) and 3 (strong staining). The percentage of positive cells was divided into five levels: 0 (≤ 5%), 1 (6–25%), 2 (26–50%), 3 (51–75%) and 4 (> 75%). The final scores for each slide were calculated by multiplying the scores for intensity and percentage. The scores less than 3 were judged to be low-expressed.

### Western blot analysis

Cells and tissues were lysed using a lysis buffer to obtain proteins, which were quantified with the BCA protein quantitative kit (Beyotime, China). Proteins were separated by SDS-PAGE and blotted onto the polyvinylidene fluoride (PVDF) membranes. The membranes were blocked with 5% DifcoTM Skim milk (BD, United States) and incubated overnight at 4 °C with specific primary antibodies and then incubated with HRP-conjugated secondary antibodies. The following antibodies were used: NCAM1/CD56 (1:1000, Proteintech, 14255-1-AP), PSA-CD56 (1:250, eBioscience, 14-9118-95), SUFU (1:1000, Cell Signaling Technology, #2520), PTCH2 (1:1000, Cell Signaling Technology, #2470), SHH (1:1000, Cell Signaling Technology, #2207), GLi1 (1:400, Cell Signaling Technology, #3538), SMO (1:1000, Cell Signaling Technology, #92981), β-catenin (1:5000, Proteintech, 66379-1-Ig), c-Myc (1:1000, Bioworld, BS2462), Cyclin D1 (1:2000, Proteintech, 60186-1-Ig), MMP2 (1:500, Senta Cruz Biotech, sc-10736), MMP7 (1:500, Proteintech, 10374-2-AP), MMP9 (1:500, AbClonal, A0289) and GAPDH (1:10,000, Proteintech, 10494-1-AP).

### Real-time quantitative PCR (RT-qPCR)

Total RNA from tissues and cells was extracted with RNAiso Plus (TAKARA BIOINC), followed by cDNA synthesis with TransScript^®^ One-Step gDNA Removal and cDNA Synthesis SuperMix (Transgen, Beijing, China). Real-time quantitative PCR was conducted with SYBR Select Master Mix (Applied Biosystems). Expression was normalized to the expression of the human housekeeping gene GAPDH. Forward and reverse primer sequences are provided in Additional file 1: Table S1.

### Survival analysis

Overall survival (OS) and progression-free survival (PFS) analysis were performed using the Kaplan–Meier Plotter website (https://kmplot.com/) [18]. We collected and collated the mRNA expression of *NCAM1* in TCGA and prognostic parameters of ccRCC patients. Univariate and multivariate Cox regression analysis was performed with SPSS 26.0 software.

### Gene functional enrichment analysis

We divided the ccRCC patients of TCGA dataset into two groups (low CD56 expression/high CD56 expression) according to the median of the expression of *NCAM1* and gene functional enrichment analysis was performed using gene set enrichment analysis (GSEA) [19].

### CCK-8 and colony formation assays

Cell proliferation capacity was determined using a Cell Count Kit-8. Briefly, cells were plated in 96-well plates at a density of 2.5 × 10^3^ cells (Caki-1 cell line) or 2 × 10^3^ cells (786-O cell line) per well in triplicate, and 10 μl of CCK-8 reagent was added into each well before incubation at 37℃ with 5% CO_2_ for two hours. The microplate reader (Thermo Fisher Scientific, United States) was used to measure the absorbance at 450 nm. In addition, 0.5 × 10^3^ cells (Caki-1 cell line) or 0.2 × 10^3^ cells (786-O cell line) per well were added in 6-well plates and incubated for 14 days or 10 days separately. The cells were fixed with 4% paraformaldehyde and stained with 1% crystal violet and there were more than 50 cells in each colony.

### Cell scratch assay

To ensure consistent photographic measurements at each time point, four equal lines were demarcated on the posterior surface of every well in a six-well plate. Subsequently, a total of ~ 5 × 10^5^ cells were seeded to each well and allowed to proliferate until reaching confluence. Subsequently, a 200 μl sterile pipette tip was used to make a gap in the monolayer. This was succeeded by a thorough rinse with phosphate-buffered saline (PBS) to remove cell debris and photographed immediately with a microscope at 100 × magnification. Following an incubation period of either 6 h or 36 h in a medium free of fetal bovine serum (FBS), the same regions were subsequently subjected to photographic documentation.

### Transwell assays

About 3 × 10^4^ cells (Caki-1 cell line) or 2 × 10^4^ (786-O cell line) cells in 200 μl of medium without FBS were added to the upper compartment of a 24-well transwell chamber (8 μm pore size polycarbonate membrane, BIOFIL, China), and 550 μl medium with 10% FBS was added to the lower compartment. After incubating for 48 h or 24 h at 37℃ with 5% CO_2_, the upper compartment was washed with PBS and fixed in 4% paraformaldehyde, then stained with 1% crystal violet. Subsequently, the chambers were washed with PBS and photographed with a microscope at 100 × magnification. For the invasion assay, the upper compartment was precoated with 40 μl Matrigel and incubated for 2 h at 37 ℃, and about 3 × 10^4^ cells in 200 μl of medium without FBS were added to the upper compartment of a 24-well transwell chamber followed by the same migration steps.

### Immunofluorescence assay

Cells were planted on glass slides at a density of about 30%. After being fixed with 4% paraformaldehyde, cells were permeabilized with 0.1% Triton-X 100 and then blocked with an immunostaining blocking solution (Beyotime, China). After that, cells were incubated overnight at 4 ℃ with primary antibody. A second antibody with fluorescent labelling was used at 37 ℃ for 1 h in the dark, and DAPI was used to stain the nucleus for 5 min. Immunofluorescence images were obtained using a laser-scanning confocal microscope. The following antibodies were used: β-catenin (1:100, Proteintech, 66379-1-Ig), CD56 (1:50, Proteintech, 14255-1-AP), PSA-CD56 (1:24, eBioscience, 14-9118-95), Alexa fluor 488-conjugated goat anti-mouse IgG (H + L) (1:100, Beyotime, A0428), FITC-conjugated goat anti-mouse IgM (H + L) (1:100, Elabscience, E-AB-1067), and TRITC-conjugated goat anti-rabbit IgG (H + L) (1:100, Proteintech, 10,066-2-AP).

### Xenograft tumor model

Animal experiments were approved by the Animal Research and Care Committee of Dalian Medical University (AEE21035). Animals were maintained under constant temperature (23–25 ℃) and humidity (45–65%) with controlled light/dark cycles (12 h/12 h). Athymic BALB/c male nude mice (4–6 weeks old) were bred and housed in the Animal Experiment Center of Dalian Medical University and randomly divided into the 786-O/Scrambled and the 786-O/CD56-KO groups, with 10 mice per group. Cells from different groups were washed and resuspended to 1 × 10^8^ cells/ml in PBS, and then 0.1 ml was subcutaneously injected into the mice back. Tumour diameter was measured with a Vernier calliper every 7 days beginning on the 7th day until the 28th day. The volumes were calculated by the formula: 1/2 × (length × width^2^). Upon the end of the experiment, after the process of euthanasia, the tumours were surgically removed and photographic documentation was obtained.

### Statistical analyses

All statistical analyses were done with GraphPad Prism 8.0.1, SPSS 26.0 and R 4.2.2 software. For sample size choice, the majority of the assays performed used either ≥ 3 donors to overcome donor variability. In all figures with histograms, data are represented using the mean ± SD. The unpaired or paired two-tailed Student’s *t* test was used for comparison between two groups. The one-way ANOVA test and two-way ANOVA test were used for three or more groups. The log-rank test was used for survival analysis. *P* < 0.05 was considered a significant value.

## Results

### The expression of CD56 was elevated in ccRCC patients and cell lines

**Fig. 1:**
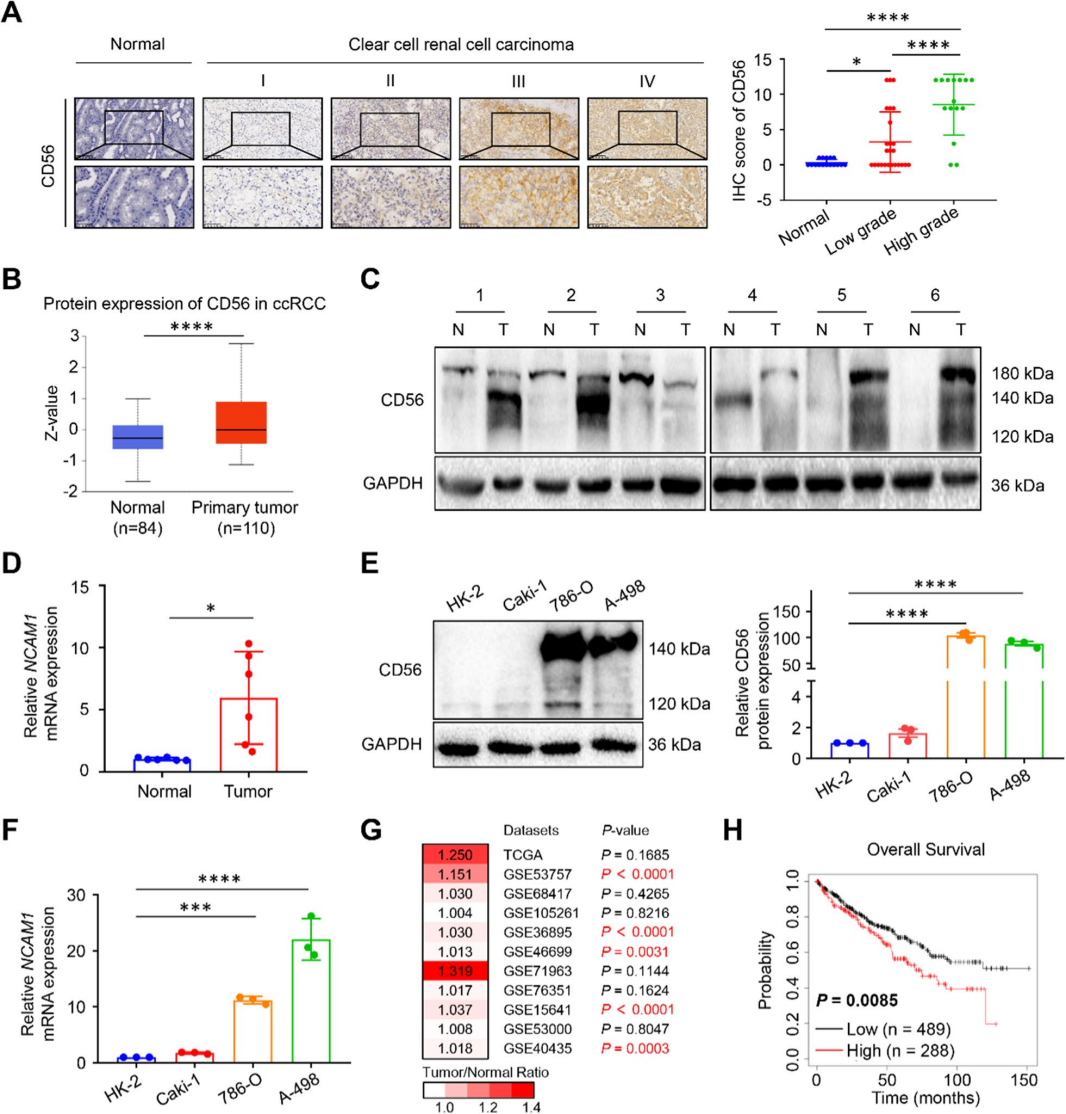
The clinical significance of elevated CD56 expression in ccRCC tissues and cell lines. **A** Immunohistochemical (IHC) analysis of CD56 expression in normal renal tissues (n = 16) and different grades of clear cell RCC specimens (n = 40). Low grade represents grade I plus grade II and high grade represents grade III plus grade IV. Representative images of CD56 immunohistochemical staining. **B** Protein expression profile of CD56 in normal renal tissues (n = 84) and primary clear cell RCC tissues (n = 110).** C** The clinical samples were collected from six patients, and the expression levels of CD56 were measured by using western blot. **D** Real-Time qPCR was conducted to determine the mRNA expression of *NCAM1* in ccRCC tissues and their paired adjacent normal tissues (n = 6). **E** Western blot was conducted to examine the protein expression of CD56 in HK-2, Caki-1, 786-O and A-498 cells. GAPDH was used as a loading control. Each experiment was repeated at least three times. **F** Real-time qPCR was used to measure the mRNA expression of *NCAM1* in the human ccRCC cell lines (Caki-1, 786-O and A-498) and human normal renal epithelial cell line (HK-2). **G** The differential expression of *NCAM1* in ccRCC and normal tissues by TCGA and GEO databases. **H** Survival curves suggested that patients with elevated *NCAM1* mRNA level showed poorer overall survival (OS) in ccRCC patients. All values represented the mean ± SD of three independent experiments. *P < 0.05, **P < 0.01, ***P < 0.001, ****P < 0.0001 by one-way ANOVA (**A, E, F**), unpaired two-tailed Student’s *t* test (**B**), paired two-tailed Student’s *t* test (**D**), or log-rank test (**H**)

To explore the expression levels and clinical significance of CD56 in ccRCC, we obtained 40 ccRCC samples with different grades and 16 normal tissues. IHC analysis showed that the expression of CD56 was significantly higher in the ccRCC patient compared to the normal one (Fig. 1A). Subsequently, we verify this result by using the Clinical Proteomic Tumor Analysis Consortium (CPTAC) database that revealed that CD56 protein expression was highly expressed in ccRCC tissues compared to the normal tissues (Fig. 1B). In a similar manner, the protein expression and mRNA levels of CD56 was significantly elevated in ccRCC tissues compared with paraneoplastic tissues (Fig. 1C, D). In addition, the expression of CD56 was positively correlated with pathological grade (*P* = 0.0014), which is in line with the protein expression of CD56 in the ccRCC CPTAC database (Table 1; Additional file 1: Fig. S1A). In vitro analysis also elucidate that, the mRNA and protein expression of CD56 were higher in ccRCC cell lines (786-O and A-498) compared to human normal renal epithelial cell line (HK-2) (Fig. 1E, F).

In-depth analysis of the CD56 and its clinical significance in ccRCC patients revealed a significant positive correlation between CD56 protein expression in ccRCC samples and clinicopathological stages (Additional file 1: Fig. S1B). Based on the TCGA and GEO databases, we analyzed the mRNA expression of *NCAM1*. Compared with normal kidney tissues, the *NCAM1* expression was higher in ccRCC tissues (Fig. 1G). Cox regression analysis for prognostic significance of the *NCAM1* expression and prognostic parameters of TCGA dataset was performed. The univariate analysis revealed that the level of *NCAM1* (*P* = 0.008), age (*P* = 0.001), grade (*P* = 3.69E-08), stage (*P* = 8.63E−12) and M (metastasis) (*P* = 7.93E−17) and T (tumor) (*P* = 1.47E−10) were associated with poor OS. Multivariate analysis showed no correlation between the expression of *NCAM1* and OS (*P* = 0.085) (Additional file 1: Table S2), and there were an association of either age (*P* = 0.001), grade (*P* = 0.002), stage (*P* = 2.26E−04), M (*P* = 5.22E−08) and T (*P* = 0.020) with OS. Furthermore, a Kaplan–Meier analysis of survival demonstrated that elevated CD56 expression was associated with shorter OS (*P* = 0.0085) (Fig. 1H) and PFS (*P* = 0.032) (Additional file 1: Fig. S1C). Overall, elevated CD56 expression was closely associated with advanced clinicopathological parameters and an unfavourable prognosis in ccRCC patients.

### CD56 knockout inhibits proliferation, migration and invasion of 786-O cells in vitro and in vivo

**Fig. 2:**
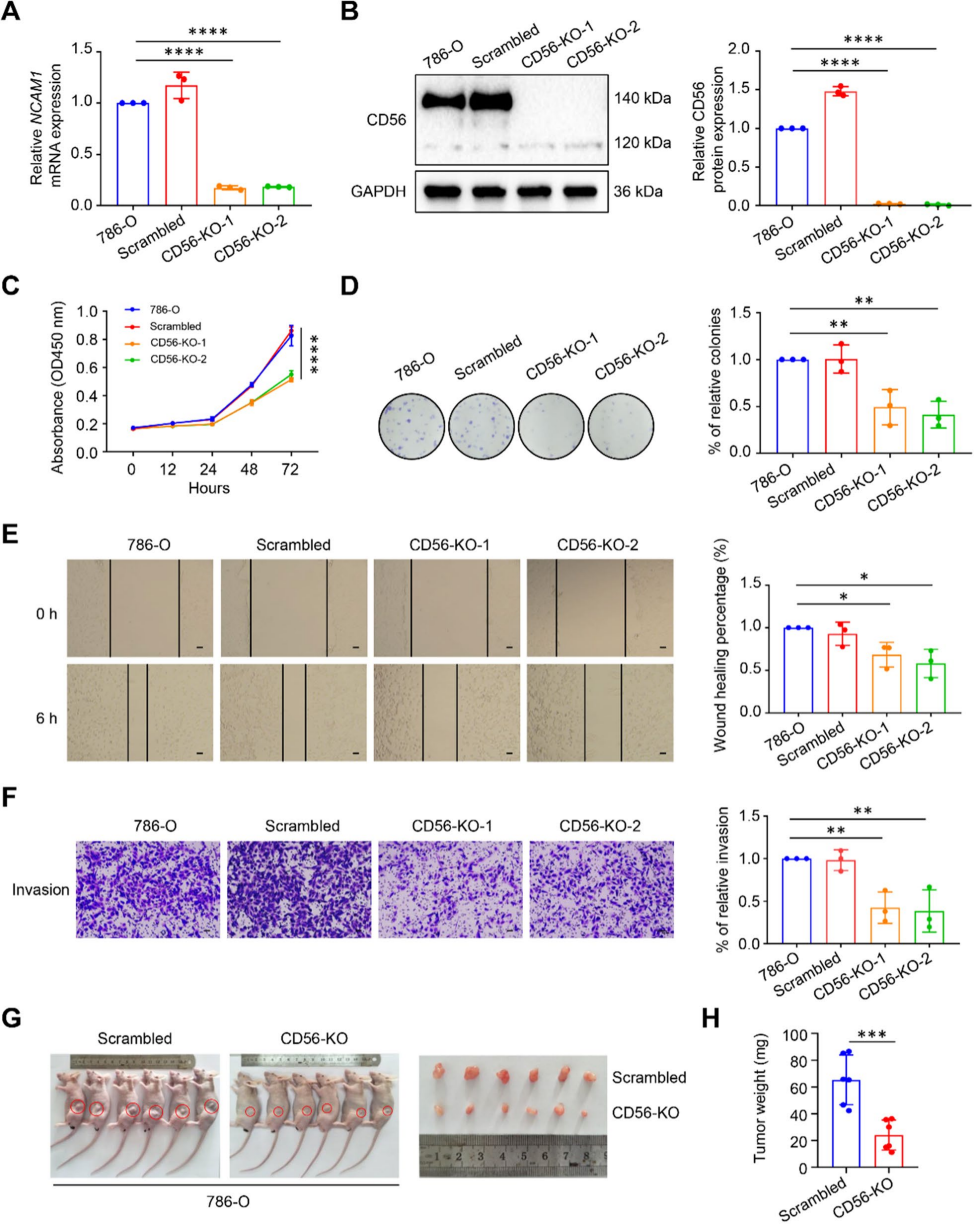
Knockout of CD56 inhibited proliferation, invasion and migration of ccRCC cells in vitro and in vivo. Real-time qPCR (**A**) and western blot (**B**) showed CD56 expression in 786-O cells using CRISPR/Cas9-mediated genome editing. **C** CCK-8 assay was performed to determine the effect of knockout-CD56 on the proliferation ability. **D** Knockout of CD56 suppressed the colony formation ability of 786-O cells compared with control cells and scrambled cells. **E** Knockout of CD56 inhibited the migration ability of 786-O cells by cell scratch assay within 6 h. **F** Transwell assay showed knockout of CD56 inhibited the 786-O cells invasion ability. **G** Morphology of subcutaneous tumors in nude mice after injection for four weeks (n = 6). **H** Average weight of tumor tissue in nude mice (n = 6). All values represented the mean ± SD of three independent experiments. Scale bars: 400 μm. *P < 0.05, **P < 0.01, ***P < 0.001, ****P < 0.0001 by one-way ANOVA (**A**, **B**, **D**, **E**, **F**), two-way ANOVA (**C**), or unpaired two-tailed Student’s *t* test (**H**)

To further investigate the potential functions of CD56 in ccRCC cell lines, we generated CD56 stable knockout (CD56-KO) cells using the human ccRCC 786-O cells by CRISPR/Cas9 genome editing technique. Compared to the control and scrambled groups, the CD56 expression levels in the CD56-KO cells were significantly reduced (Fig. 2A, B). CCK-8 assay result showed that knocking out CD56 inhibited the growth and proliferation of 786-O cells (Fig. 2C). Moreover, the clone formation rate of CD56-KO cells was significantly lower than that of the control group (Fig. 2D). Cell scratch assay and transwell assays were also performed to analyze cell migration and invasion capabilities in vitro. The result of the cell scratch assay showed that the migration rate of CD56-KO cells decreased within 6 h compared with the control and scrambled groups (Fig. 2E). Consistent with the result of the wound healing assay, the transwell assays showed that the number of cells that migrated and invaded the lower chamber within 24 h in the CD56-KO group was significantly reduced (Fig. 2F; Additional file 1: Fig. S2A).

In light of the significant importance of CD56 in ccRCC, we conducted additional in vivo investigations to elucidate the impact of CD56 knockout on ccRCC development. Specifically, we administered subcutaneous injections of scrambled cells and CD56-KO cells into athymic male nude mice. Our findings indicated that the tumour weight and volume (Fig. 2G, H; Additional file 1: Fig. S2B) were comparatively lower in the CD56-KO-injected nude mice compared to those injected with scrambled cells. The western blot analysis revealed a notable decrease in the expression level of CD56 in the nude mice injected with CD56-KO compared to those injected with scrambled, as depicted in Additional file 1: Fig. S2C. Therefore, the findings of this study demonstrated that the elimination of CD56 in human ccRCC 786-O cells effectively suppressed theirs in vitro and in vivo proliferation, migration, and invasion capabilities.

### Knockout of CD56 inhibits the hedgehog and Wnt/β-catenin signaling pathways in ccRCC

**Fig. 3:**
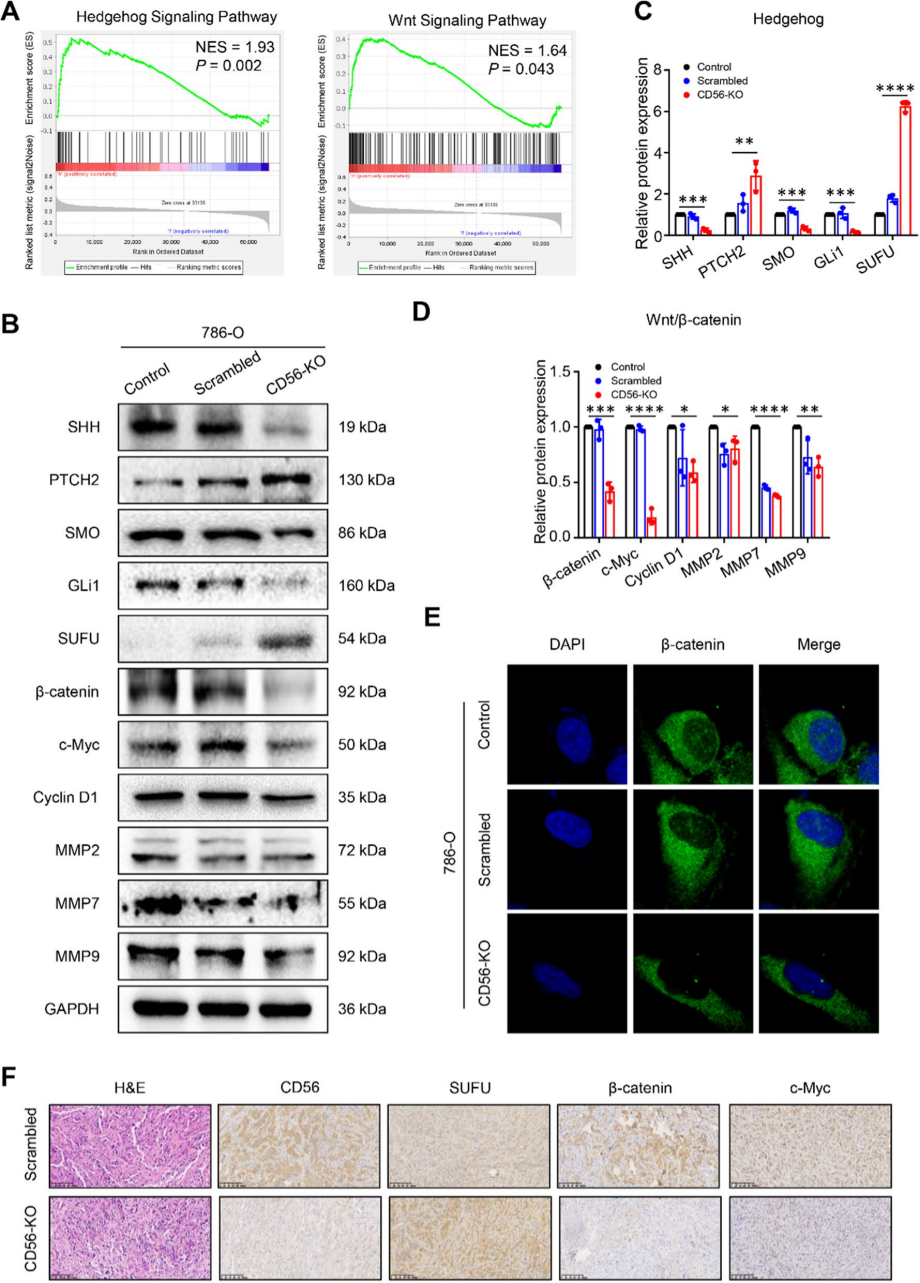
Knockout of CD56 inhibited Hedgehog and Wnt/β-catenin signaling pathways in 786-O cells.** A** Pathway enrichment analysis according to CD56 expression in ccRCC by GSEA. **B** Representative images of the protein of Hedgehog and Wnt/β-catenin signaling pathways measured by western blot. **C** Grayscale analysis statistics of protein expression levels of Hedgehog signaling pathway. **D** Grayscale analysis statistics of protein expression levels of Wnt/β-catenin signaling pathway. **E** Detection of β-catenin expression in cytoplasm and nucleus by immunofluorescence assay. **F** H&E, CD56, SUFU, β-catenin and c-Myc staining images of subcutaneous tumour tissue in nude mice. All values represented the mean ± SD of three independent experiments. *P < 0.05, **P < 0.01, ***P < 0.001, ****P < 0.0001 by one-way ANOVA (**C**, **D**)

To elucidate the potential molecular mechanisms of CD56 mediating the malignant phenotype of ccRCC cells, we conducted gene enrichment analysis by GSEA. This analysis allowed us to investigate the signaling pathways that were associated with CD56 in ccRCC. According to the findings presented in Fig. 3A and Additional file 1: Fig. S3A, CD56 is involved in several crucial signaling pathways, such as Hedgehog, Wnt/β-catenin, T cell receptor, ECM receptor, and calcium signaling pathways. Notably, the expression of CD56 exhibited a significant correlation with the Hedgehog and Wnt/β-catenin signaling pathways. Recent research has indicated that the sonic hedgehog (SHH) pathway exhibits activity in ccRCC, and the administration of drugs that inhibit this pathway has been found to decrease the migratory capacity of ccRCC cells [20]. Furthermore, it has been observed that the Hedgehog signaling pathway and Wnt/β-catenin signaling pathway possess the ability to mutually regulate and communicate with one another bidirectionally [21]. Consequently, an analysis was conducted on the protein expression of pathway-related proteins in both CD56-knockout 786-O cells and control cells (Fig. 3B, C, D). The findings of this analysis revealed that the depletion of CD56 resulted in reduced protein levels of β-catenin, c-Myc, Cyclin D1, and matrix metalloproteinases (MMPs) within the Wnt/β-catenin signaling pathway. Additionally, it led to decreased levels of SHH, SMO, and GLi1 within the Hedgehog signaling pathway. Conversely, the expression of inhibitory proteins, namely PTCH2 and SUFU, within the Hedgehog signaling pathway was found to be increased. Furthermore, the immunofluorescence findings indicated a reduction in the expression of β-catenin in both the cytoplasm and nucleus of the CD56-KO cells when compared to the cells in the control and scrambled groups (Fig. 3E). Consistently, the immunohistochemical analysis results of ccRCC tumour tissue in nude mice showed that, compared with the scrambled group, the expression levels of CD56, β-catenin and c-Myc in the CD56-KO group were significantly decreased, while the expression level of SUFU was significantly increased (Fig. 3F). Additionally, compared with the scrambled group, the expression levels of SHH, GLi1, β-catenin, c-Myc and Cyclin D1 in the CD56-KO group were significantly reduced, while the protein expression of PTCH2 and SUFU was significantly increased (Additional file 1: Fig. S3B). All of these findings together pointed to the possibility that CD56 deletion in ccRCC might reduce the activity of the hedgehog and Wnt/β-catenin signaling pathways.

### PSA-CD56 promotes proliferation, migration and invasion of Caki-1 cells via Hedgehog and Wnt/β-catenin signaling pathways in vitro

**Fig. 4:**
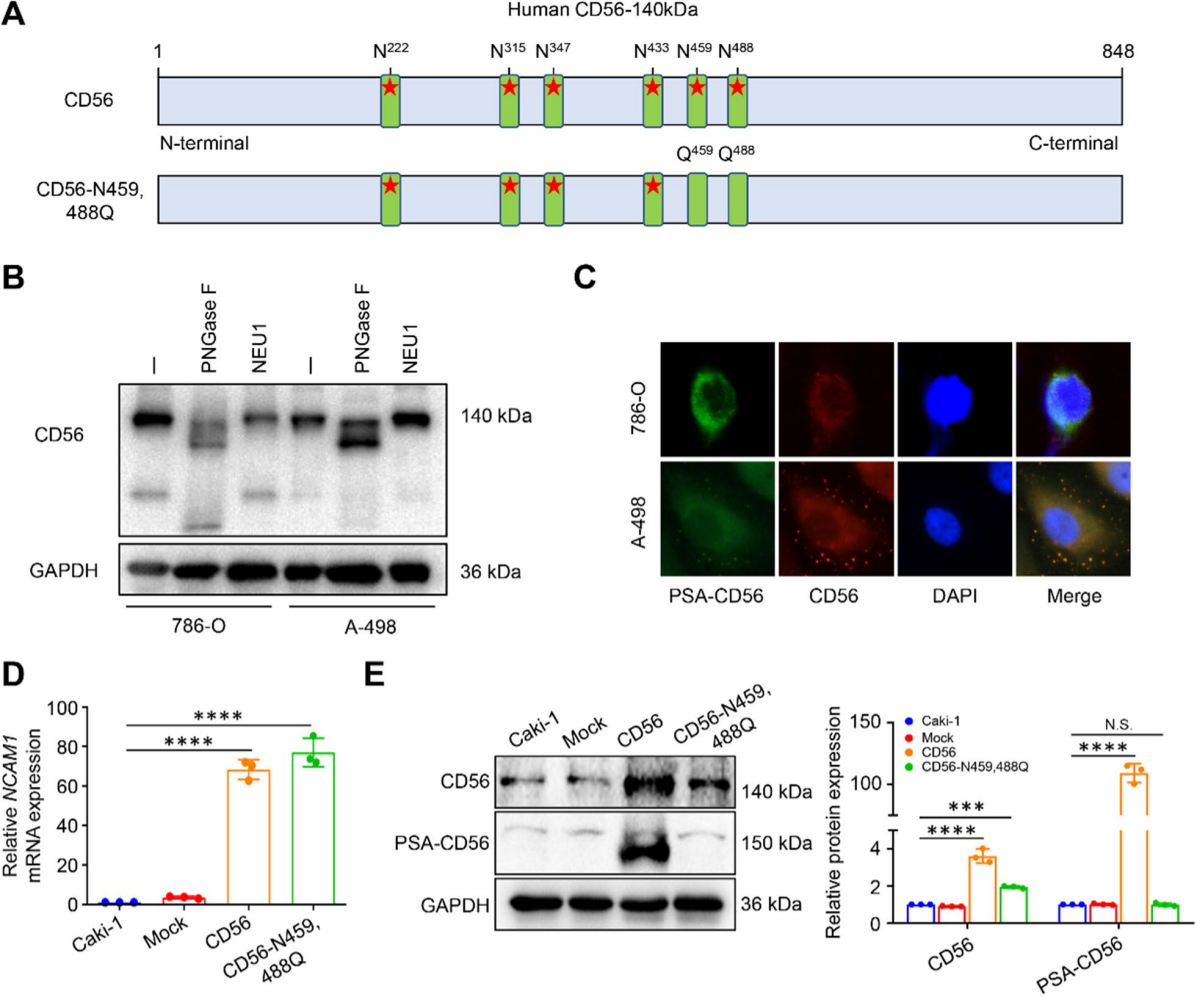
CD56 is polysialylated in ccRCC cells. **A** Primary structure diagram of CD56 and CD56-N459,488Q. The pentagrams represent the *N*-glycosylation sites. N represents asparagine and Q represents glutamine. **B** Polysialylation of CD56. 786-O and A-498 cells were lysed and then treated with or without PNGase F and NEU1 for 32 h in 37 ℃. **C** Co-localization of CD56 and PSA-CD56 in 786-O cells. Cells were stained with anti-CD56 (1:50, red), anti-PSA-CD56 (1:24, green), DAPI (blue) and then subjected to confocal fluorescence microscopy. Overexpression of CD56 and CD56-N459,488Q in Caki-1 cells was analyzed by Real-time qPCR (**D**) and western blot (**E**). ***P < 0.001, ****P < 0.0001, N.S. represents no statistical significance by one-way ANOVA (**D**, **E**)

CD56 possesses a total of six potential *N*-glycosylation sites namely N222, N315, N347, N433, N459 and N488 (Fig. 4A, upper) located on its third, fourth, and fifth Ig-like domains. Notably, the CD56 gene undergoes translation and transcription at the terminus of the glycan chain found on the fifth Ig-like domain. This process is facilitated by polysialyltransferases, resulting in the formation of polysialylated CD56, commonly referred to as PSA-CD56 [13, 14, 22]. In order to confirm the *N*-glycosylation on CD56, deglycosylation was performed using a peptide-*N*-glycosidase from Flavobacterium meningosepticum (PNGase F). The amide link between the first saccharide N-acetylglucosamine (GlcNAc) is cleaved by PNGase F, while the terminal sialic acids are cleaved by neuraminidase 1 (NEU1). Our Results indicate that CD56 is an *N*-glycosylated protein (Fig. 4B**)**. By using immunofluorescence assays, we found that CD56 was polysialylated in 786-O and A-498 cells (Fig. 4C). For the purpose of investigating the potential impact of polysialic acid modification on the malignant progression of ccRCC, we employed molecular cloning techniques to construct overexpression vectors for CD56 and CD56-N459,488Q and the latter mutated the asparagine (Asn, N) to glutamine (Gln, Q) at 459 and 488 sites of CD56 (Fig. 4A, lower). Finally, we checked for CD56 mRNA and protein expression levels which were significantly increased in CD56-OE and CD56-N459,488Q-OE groups compared to the control and mock groups (Fig. 4D, E). These findings indicate that CD56 is polysialylated in ccRCC cells and we successfully established a stable overexpression of CD56 and CD56-N459,488Q Caki-1 cell lines.

**Fig. 5:**
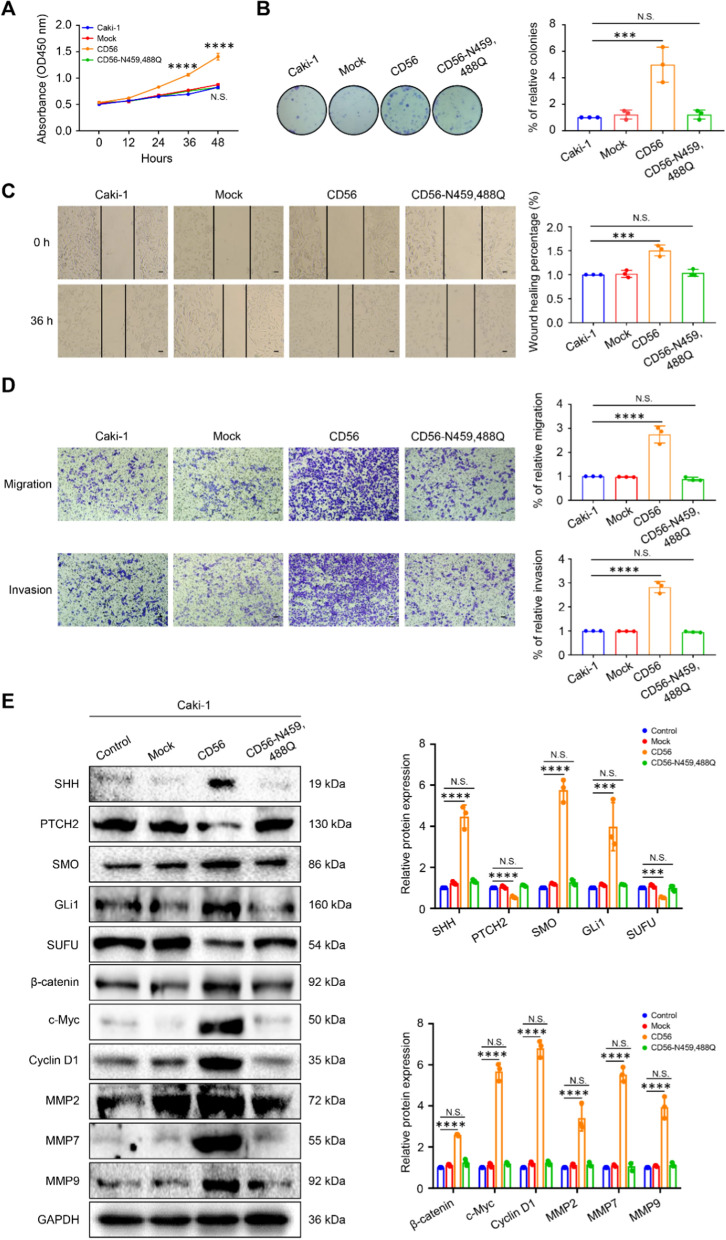
PSA-CD56 promoted proliferation, invasion and migration of ccRCC cells in vitro. **A** The proliferation ability of Caki-1 cells with stably overexpression-CD56 and CD56-N459,488Q was determined by CCK-8 assay. **B** The colony formation ability of cells was evaluated by colony formation assay. **C** The migration ability of overexpression of CD56 and CD56-N459,488Q in Caki-1 cells by cell scratch assay within 36 h. **D** Transwell assays for the effects of overexpression CD56 and CD56-N459,488Q on migration and invasion ability of Caki-1 cells. **E** Representative images of the protein of Hedgehog and Wnt/β-catenin signaling pathways in Caki-1 cells with stably overexpression-CD56 and CD56-N459,488Q measured by western blot. Results were expressed as the mean ± SD of three independent experiments. Scale bars: 400 μm. **P < 0.01, ***P < 0.001, ****P < 0.0001, N.S. represents no statistical significance by two-way ANOVA (**A**), or one-way ANOVA (**B**, **C**, **D**, **E**)

To investigate the function of PSA-CD56 in the malignant phenotypes of ccRCC cells, CCK-8 and colony formation assays showed that overexpression of CD56 promoted the proliferation and colony formation ability of Caki-1 cells, but there was no obvious difference between the CD56-N459,488Q-OE and control groups (Fig. 5A, B). In addition, cell scratch assay (Fig. 5C) and transwell assays (Fig. 5D) showed that the migration rate and the number of cells invading the lower chamber were significantly increased in the CD56-OE group, while the CD56-N459,488Q-OE group was no distinct difference with the control group. Taken together, these results demonstrate that PSA-CD56 overexpression of Caki-1 cells promotes their proliferation, migration and invasive ability in vitro.

Overexpression of CD56 increased the protein expression levels of β-catenin, c-Myc, Cyclin D1, and MMPs in Wnt/β-catenin signaling pathway and SHH, SMO and GLi1 in the Hedgehog signaling pathway, and decreased the expression of PTCH2 and SUFU in the Hedgehog signaling pathway. However, the protein expression of CD56-N459,488Q-OE group had no obvious change compared with control group (Fig. 5E). Taken together, these results indicate that overexpression-PSA-CD56 promoted the proliferation, migration and invasion of ccRCC cells via Hedgehog and Wnt/β-catenin signaling pathways.

### JK184 and Prodigiosin inhibit the Hedgehog and Wnt/β-catenin signaling pathways in CD56-OE cells

**Fig. 6:**
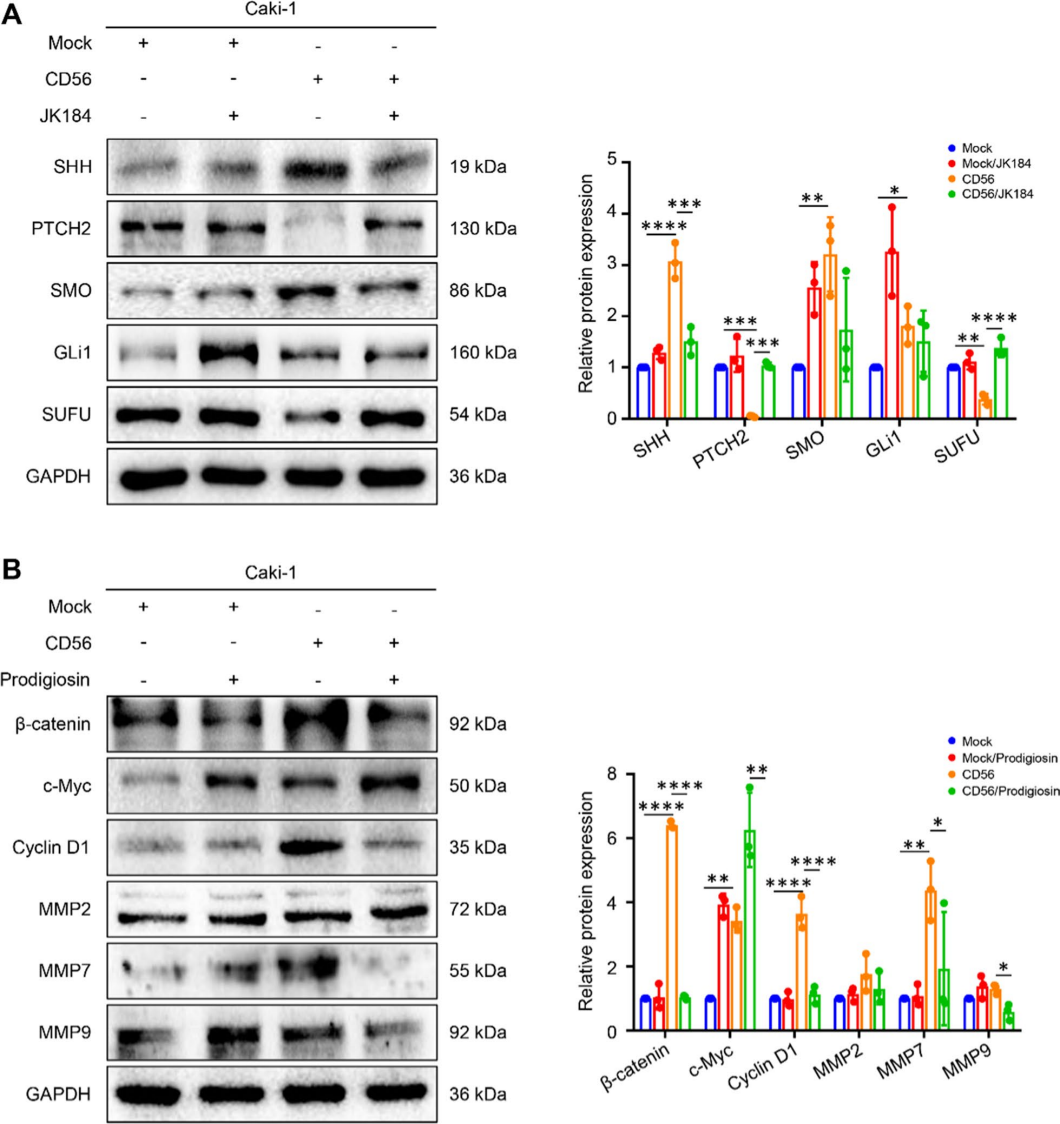
JK184 and Prodigiosin inhibit the Hedgehog and Wnt/β-catenin signaling pathways in CD56-OE cells. **A** Cells were pretreated with 10 nM JK184 for 48 h. Representative images showing protein expression levels of Hedgehog signaling pathway by western blot. **B** Cells were pretreated with 500 nM Prodigiosin for 48 h. Representative images showing protein expression levels of Wnt/β-catenin signaling pathway by western blot. *P < 0.05, **P < 0.01, ***P < 0.001, ****P < 0.0001 by one-way ANOVA (**A**, **B**)

To further explore the relationship between CD56 and Hedgehog and Wnt/β-catenin signaling pathways, the CD56-OE group was treated with Hedgehog inhibitor JK184 and β-catenin inhibitor Prodigiosin, respectively. Figure 6A illustrates that JK184 treatment significantly decreased the expression levels of SHH, SMO, and GLi1 in the CD56-OE group. Conversely, it increased the expression levels of PTCH2 and SUFU. In addition, Prodigiosin treatment reduced the expression levels of β-catenin, Cyclin D1 and MMP7 in the CD56-OE group. However, there was no difference in the levels of MMP2 and MMP9 between the CD56-OE group and CD56-OE/Prodigiosin group, and the expression of c-Myc was increased with Prodigiosin treatment (Fig. 6B). These results demonstrate that JK184 and Prodigiosin treatments can effectively restore the expression levels of relevant signaling pathway proteins in the CD56-OE group.

## Discussion

CD56 is a glycoprotein that exists on the surface of various cells and belongs to the immunoglobulin superfamily [23]. It plays a vital role in the growth and development of the nervous system as well as in facilitating cell–cell adhesion [24, 25]. Additionally, CD56 can influence the infiltration, invasion and metastasis of malignant tumours [5, 10, 26]. A previous study has shown that CD56 is a sensitive and diagnostic immunohistochemical marker for ovarian sex chromosome tumours [27]. In this study, we aimed to investigate the role of CD56 in clear cell renal cell carcinoma, as its specific contribution to the complex process of cancer progression has not been extensively examined. By evaluating CD56 in the context of ccRCC, we sought to shed light on its potential implications and provide a deeper understanding of its involvement in this particular type of cancer.

We have demonstrated that the expression of CD56 was significantly increased in ccRCC compared with normal renal epithelium, which was verified on patient tissues and cell lines. The aforementioned findings were additionally corroborated by the CPTAC database. Following this, it was discovered that the increased expression of CD56 was associated with the clinical advancement and unfavourable prognosis of ccRCC patients. This implies that CD56 may play a role in the initiation and progression of ccRCC. As a widely used neuroendocrine marker, CD56 is highly sensitive to neuroendocrine tumours [28–30], speculating that ccRCC may be a tumour with neuroendocrine characteristics, and CD56 has the potential to be a marker for assisting the diagnosis of ccRCC.

Furthermore, this study demonstrated a significant decrease in the proliferation, migration and invasion abilities of 786-O-CD56-KO cells were reduced compared with 786-O cells. Interestingly, CD56 undergoes post-translational modifications during maturation, leading to the formation of polysialylated CD56, known as PSA-CD56. Previous research has predominantly focused on PSA-CD56's involvement in the development of the nervous system, specifically in processes like neuronal migration, cytokine response [31], and cell contact-dependent differentiation [32]. Moreover, the involvement of PSA-CD56 in immunological responses is expected and it also shows the regulatory function of CD56-dependent synaptic plasticity in mammals highlighting its multifunctional role [33]. However, there is no report that the influence of PSA-CD56 on the malignant biological behaviours of ccRCC cells. In order to investigate the potential role of polysialylated CD56 in facilitating the malignant progression of ccRCC cells, we generated a mutated variant of CD56 by substituting asparagine (Asn, N) residues with glutamine (Gln, Q) at positions 459 and 488 (referred to as CD56-N459,488Q). In comparison to CD56-OE cells, CD56-N459,488Q-OE cells do not exhibit the ability to induce proliferation and migration in ccRCC. This observation suggests that polysialylated CD56 plays a role as a cancer-promoting factor in ccRCC. However, further investigation is warranted to explore the mechanisms by which PSA-CD56 contributes to the initiation and progression of ccRCC.

Accumulating evidence suggests that aberrant glycoproteins of tumour cells are involved in the regulation of signaling pathways related to cell growth [34], proliferation and adhesion [35, 36]. Nuclear translocation of β-catenin in the Wnt/β-catenin signaling pathway increases the expression levels of c-Myc and MMPs, thereby accelerating cancer progression [37, 38]. β-catenin protein prevents hepatocellular carcinoma cell apoptosis by triggering EMT and up-regulating MMPs levels and enhances the migration ability of hepatocellular carcinoma cells [39]. Recent studies have presented the Von Hippel-Lindau (VHL), a tumor suppressor gene strongly associated with renal cell carcinoma, as a β-catenin target [40]. This confirms that Wnt/β-catenin signaling is likely playing a central role during renal carcinoma development. In a typical Hedgehog signal transduction process, without a Hedgehog ligand, PTCH will block the activity of the pathway by inhibiting the activity of the transmembrane G protein-coupled receptor (GPCR)-family protein Smoothened (SMO) [41]. However, when Hedgehog ligand binds to PTCH, SMO is activated and released [42], which triggers the activation and release of glioma-related genes (GLi) transcription factors from the protein Suppressor of Fused (SUFU) [43], and then combined with the target gene promoter to activate the transcription of the Hedgehog target gene [44, 45]. Various studies have demonstrated that the aberrant activation of the Hedgehog signaling pathway can facilitate the migration and invasion of metastatic cancers, including basal cell carcinoma [46, 47], prostate cancer [48], oesophagal cancer [49], pancreatic cancer [50] and other malignant tumours [51]. Our study found that after overexpression of PSA-CD56, the expression levels of SHH, SMO, GLi1, β-catenin, c-Myc, Cyclin D1 and MMPs were increased to different degrees and the protein expression levels of PTCH2 and SUFU was decreased. This suggested that PSA-CD56 activated the Hedgehog and Wnt/β-catenin signaling pathways and promoted the expression levels of downstream molecules, and the use of the Hedgehog inhibitor JK184 and the β-catenin inhibitor Prodigiosin further confirmed our results. Overall, overexpression of PSA-CD56 promoted the proliferation, migration and invasion of ccRCC cells via the Hedgehog and Wnt/β-catenin signaling pathways. However, it is to be further elucidated which upstream molecule acts on the signaling pathway and how it mediates the action of key target proteins in the pathway.

## Conclusion

**Fig. 7:**
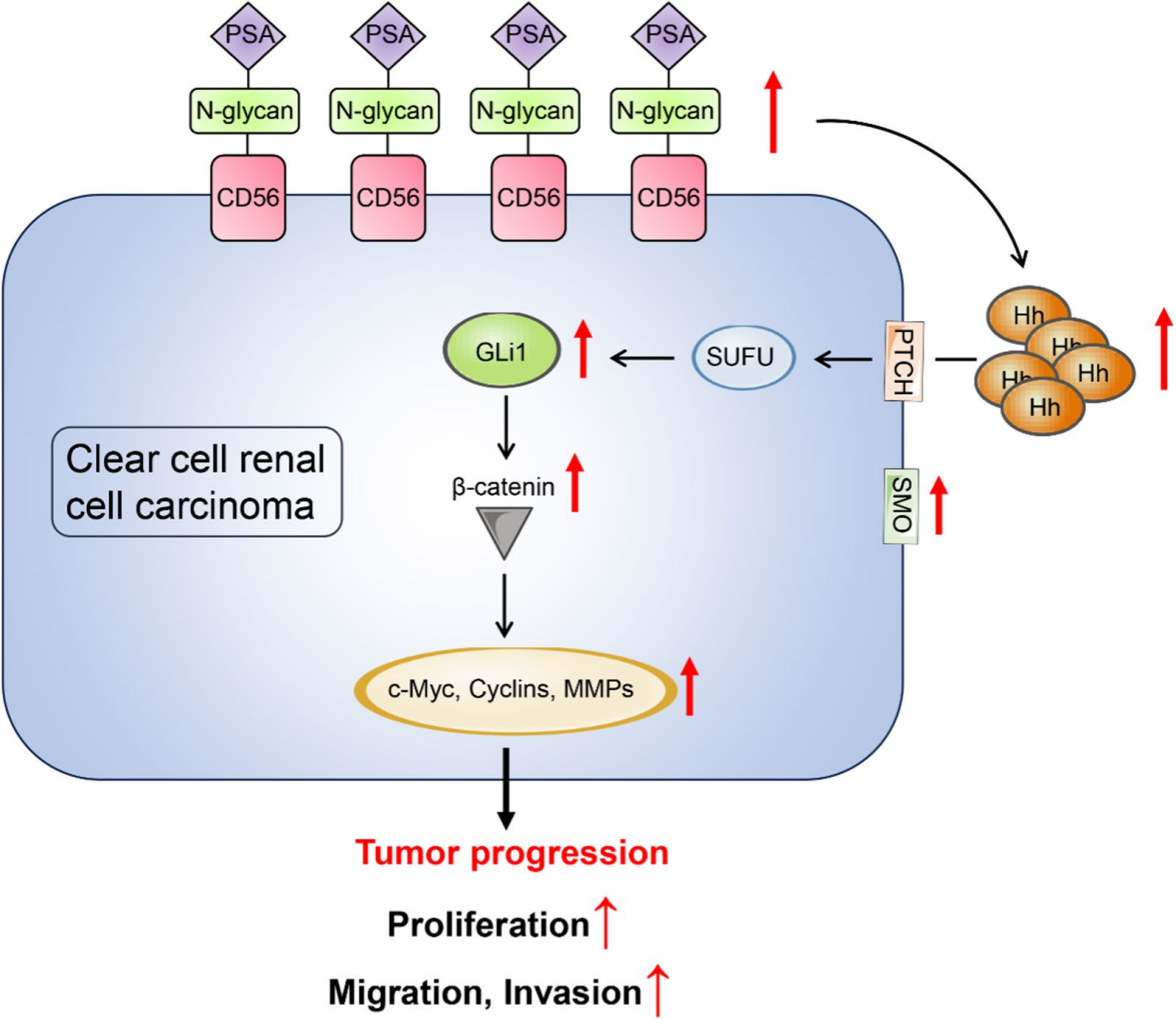
The molecular mechanisms by which CD56 polysialylation promotes the occurrence of malignant progression in clear cell renal cell carcinoma. PSA-CD56 overexpression in ccRCC enhances malignant progression via Hedgehog and Wnt/β-catenin pathways

In our groundbreaking study, we discovered that CD56 exhibited high expression levels in both human ccRCC tissues and cell lines, with a positive correlation to pathological grades. Furthermore, we found that CD56 was polysialylated in ccRCC cells. Through knockout and overexpression experiments, we observed that CD56 and its polysialylated form (PSA-CD56) modulated the expression of key molecules in the Hedgehog and Wnt/β-catenin signaling pathways. Consequently, these manipulations resulted in the reduction or enhancement of proliferation, migration, and invasion capabilities of ccRCC cells, both in vitro and in vivo (Fig. 7). Altogether, our findings suggest that overexpression of PSA-CD56 contributes to ccRCC tumorigenesis, positioning it as a potential diagnostic and therapeutic target for ccRCC.

### Supplementary Information

**Additional file 1: Figure S1 A** The protein expression of CD56 was significantly correlated with grades of ccRCC, patients who were in more advanced grades tended to express higher expression of CD56. **B** The protein expression of CD56 was significantly correlated with stages of ccRCC, patients who were in more advanced stages tended to express higher expression of CD56. **C** Survival curves suggested that patients with elevated *NCAM1* mRNA levels showed shorter progression-free survival (PFS) in ccRCC patients. *P < 0.05, **P < 0.01, ***P < 0.001 by one-way ANOVA (**A** and** B**) or log-rank test (**C**). **Figure S2 A** Transwell assays showed knockout of CD56 inhibited the migration ability of 786-O cells. **B** Tumor volume in nude mice was calculated using the formula: 1/2 × (length × width^2^) (n = 6). **C** Western blot was used to determine the expression level of CD56 (n = 3). **P < 0.01, ***P < 0.001, ****P < 0.0001 by one-way ANOVA (**A**), two-way ANOVA (**B**), or unpaired two-tailed Student’s *t* test (**C**). **Figure S3 A** Pathway enrichment analysis according to CD56 expression in ccRCC by GSEA. **B** Western blot was used to detect the expression of proteins of Hedgehog and Wnt/β-catenin signaling pathways in nude mice. Results were expressed as the mean ± SD of three independent experiments. **P < 0.01, ****P < 0.0001 by unpaired two-tailed Student’s *t* test (**B**). **Table S1: **Primers design. **Table S2**: Univariate and multivariate overall survival analysis for *NCAM1* in TCGA database.

## Data Availability

All data needed to evaluate the conclusions in the paper are present in the paper and/or the Additional files. Additional data related to this paper may be requested from the authors.

## Abbreviations

PSA-CD56: Polysialylated CD56
ccRCC: Clear cell renal cell carcinoma
IHC: Immunohistochemistry
RCC: Renal cell cancer
pRCC: Papillary RCC
chRCC: Chromophobe RCC
uRCC: Unclassified RCC
OS: Overall survival
PFS: Progression-free survival
NCAM1: Neural cell adhesion molecule-1
NK cell: Natural killer cell
EMT: Epithelial-to-mesenchymal transition
KO: Knockout
OE: Overexpression
TCGA: The Cancer Genome Atlas
GEO: Gene Expression Omnibus
STR: Short tandem repeat
DAB: Diaminobenzidine
RT-qPCR: Real-time quantitative PCR
GSEA: Gene set enrichment analysis
SHH: Sonic hedgehog
MMPs: Matrix metalloproteinases
CPTAC: Clinical Proteomic Tumor Analysis Consortium
GPCR: G protein-coupled receptor
HR: Hazard ratio
CI: Confidence interval
M: Metastasis
T: Tumor
N: Node
