# Supplementary material for: CD56 polysialylation promotes the tumorigenesis and progression via the Hedgehog and Wnt/β-catenin signaling pathways in clear cell renal cell carcinoma
Source: Cancer Cell Int. 2023 Dec 12;23:319. doi: 10.1186/s12935-023-03165-5 (PMC10717404; doi:10.1186/s12935-023-03165-5)
Supplement: Supplementary file 1 — Additional file 1: Figure S1 A The protein expression of CD56 was significantly correlated with grades of ccRCC, patients who were in more advanced grades tended to express higher expression of CD56. B The protein expression of CD56 was significantly correlated with stages of ccRCC, patients who were in more advanced stages tended to express higher expression of CD56. C Survival curves suggested that patients with elevated NCAM1 mRNA levels showed shorter progression-free survival (PFS) in ccRCC patients. *P < 0.05, **P < 0.01, ***P < 0.001 by one-way ANOVA (A and B) or log-rank test (C). Figure S2 A Transwell assays showed knockout of CD56 inhibited the migration ability of 786-O cells. B Tumor volume in nude mice was calculated using the formula: 1/2 × (length × width2) (n = 6). C Western blot was used to determine the expression level of CD56 (n = 3). **P < 0.01, ***P < 0.001, ****P < 0.0001 by one-way ANOVA (A), two-way ANOVA (B), or unpaired two-tailed Student’s t test (C). Figure S3 A Pathway enrichment analysis according to CD56 expression in ccRCC by GSEA. B Western blot was used to detect the expression of proteins of Hedgehog and Wnt/β-catenin signaling pathways in nude mice. Results were expressed as the mean ± SD of three independent experiments. **P < 0.01, ****P < 0.0001 by unpaired two-tailed Student’s t test (B). Table S1: Primers design. Table S2: Univariate and multivariate overall survival analysis for NCAM1 in TCGA database. [file 12935_2023_3165_MOESM1_ESM.docx]

**Table S1: Primers design.**

| **Primer** | **Forward** | **Reverse** |
| --- | --- | --- |
| *NCAM1* | 5’-CCGATTCATAGTCCTGTCCAA-3’ | 5’-ATAAGTGCCCTCATCTGTTTTCT-3’ |
| *GAPDH* | 5’-TCCAAAATCAAGTGGGGCGA-3’ | 5’-AAATGAGCCCCAGCCTTCTC-3’ |

**Table S2: Univariate and multivariate overall survival analysis for *NCAM1* in TCGA database.**

| **Variable** | **Univariate analysis** |  | **Multivariate analysis** |  |
| --- | --- | --- | --- | --- |
|  | **HR (95% CI)** | ***P*-value** | **HR (95% CI)** | ***P*-value** |
| *NCAM1* expression | 1.181 (1.045-1.335) | **0.008** | 1.118 (0.985-1.269) | 0.085 |
| Age | 1.656 (1.225-2.239) | **0.001** | 1.689 (1.246-2.289) | **0.001** |
| Gender | 0.948 (0.694-1.296) | 0.740 | - | - |
| Grade | 2.642 (1.869-3.733) | **3.69E-08** | 1.769 (1.230-2.544) | **0.002** |
| Stage | 3.369 (2.377-4.774) | **8.63E-12** | 6.001 (2.316-15.550) | **2.26E-04** |
| M | 3.737 (2.741-5.096) | **7.93E-17** | 2.523 (1.808-3.521) | **5.22E-08** |
| T | 2.998 (2.143-4.195) | **1.47E-10** | 0.339 (0.136-0.844) | **0.020** |
| N | 0.917 (0.679-1.238) | 0.572 | - | - |

TCGA, The Cancer Genome Atlas; HR, hazard ratio; CI, confidence interval; M, metastasis; T, tumor; N, node.

**Figure S1**


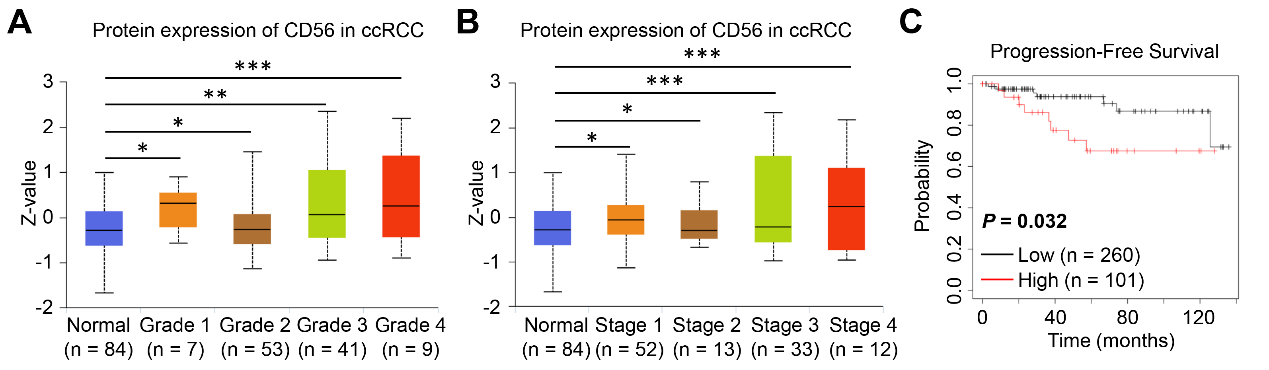


**Fig. S1** **A** The protein expression of CD56 was significantly correlated with grades of ccRCC, patients who were in more advanced grades tended to express higher expression of CD56. **B** The protein expression of CD56 was significantly correlated with stages of ccRCC, patients who were in more advanced stages tended to express higher expression of CD56. **C** Survival curves suggested that patients with elevated *NCAM1* mRNA levels showed shorter progression-free survival (PFS) in ccRCC patients. **P* < 0.05, ***P* < 0.01, ****P* < 0.001 by one-way ANOVA (**A** and **B**) or log-rank test (**C**).

**Supplementary Figure 2**


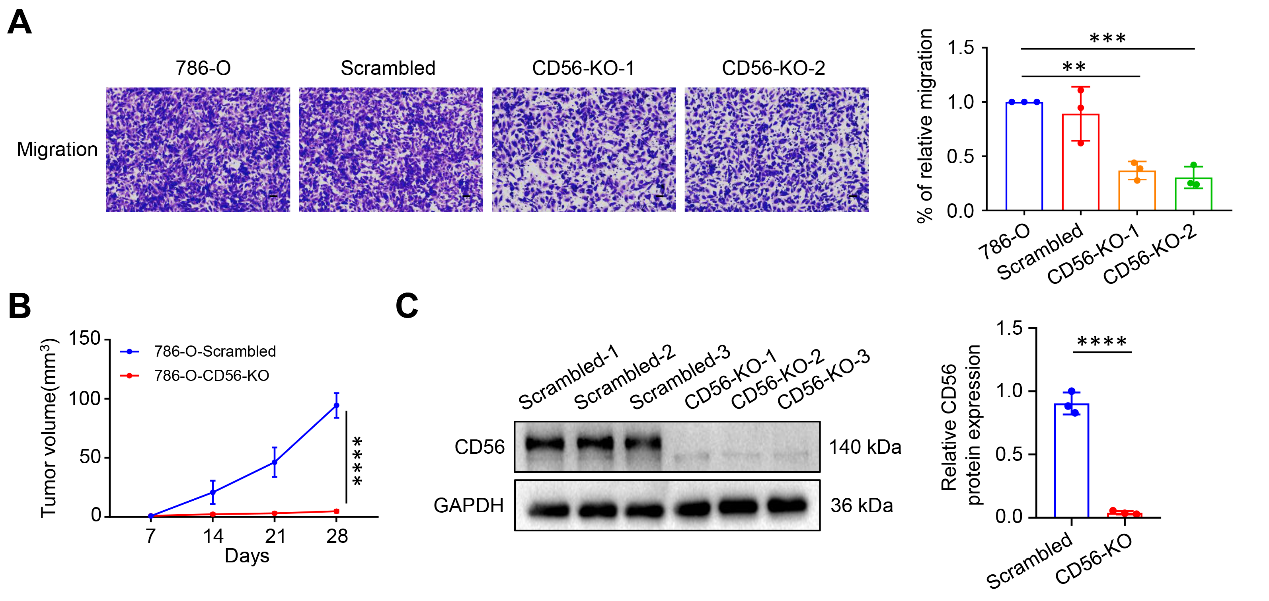


**Fig. S2 A** Transwell assays showed knockout of CD56 inhibited the migration ability of 786-O cells. **B** Tumor volume in nude mice was calculated using the formula: 1/2×(length×width^2^) (n = 6). **C** Western blot was used to determine the expression level of CD56 (n = 3). ****P* < 0.001, *****P* < 0.0001 by one-way ANOVA (**A**), two-way ANOVA (**B**), or unpaired two-tailed Student’s *t* test (**C**).

**Figure S3**


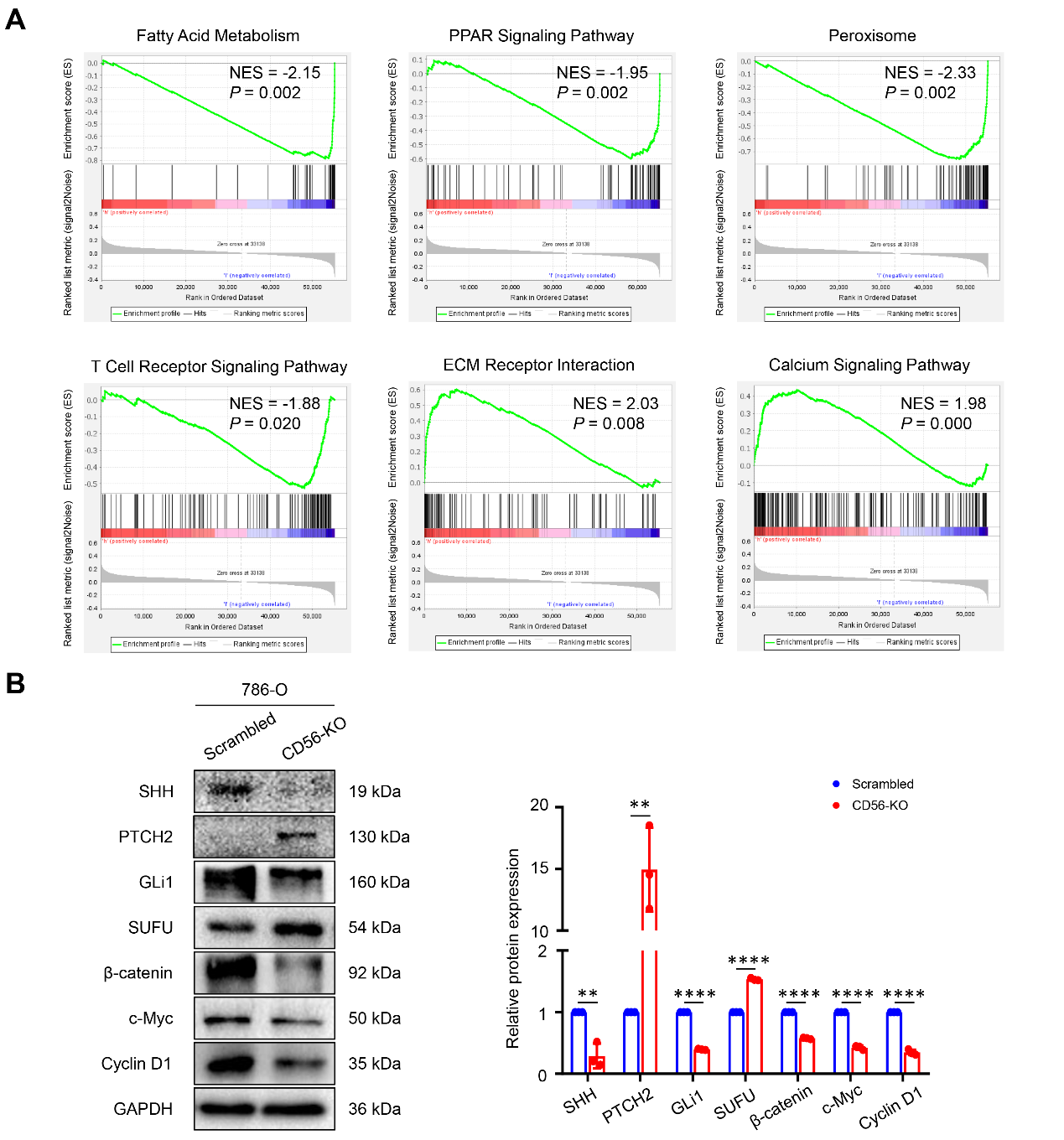


**Fig. S3 A** Pathway enrichment analysis according to CD56 expression in ccRCC by GSEA. **B** Western blot was used to detect the expression of proteins of Hedgehog and Wnt/β-catenin signaling pathways in nude mice. Results were expressed as the mean ± SD of three independent experiments. ***P* < 0.01, *****P* < 0.0001 by unpaired two-tailed Student’s *t* test (**B**).
